# Combined cardio-protective ability of syringic acid and resveratrol against isoproterenol induced cardio-toxicity in rats via attenuating NF-kB and TNF-α pathways

**DOI:** 10.1038/s41598-020-59925-0

**Published:** 2020-02-25

**Authors:** Manjunatha S., Althaf Hussain Shaik, Maruthi Prasad E., Suliman Yousef Al Omar, Altaf Mohammad, Lakshmi Devi Kodidhela

**Affiliations:** 1 Department of Biochemistry, Sri Krishnadevaraya University, Anantapur, Andhra Pradesh India; 2 Central Laboratory, Department of Zoology, College of Science, King Saud University, Riyadh, Saudi Arabia; 3 Shenzhen key of Laboratory of Translational medicine of Tumor, A7, 451, Department of Cell Biology and Genetics, Shenzhen University Health Science Centre, Shenzhen, Guangdong China; 4 Doping Research Chair, Department of Zoology, College of Science, King Saud University, Riyadh, Saudi Arabia; 5 Central Laboratory, Department of Chemistry, College of Science, King Saud University, Riyadh, Saudi Arabia

**Keywords:** Diagnostic markers, Toxicology

## Abstract

The study was conducted to evaluate the cardio-protective activity of combination (COMB) of syringic acid (SA) and resveratrol (RV) against isoproterenol (ISO) induced cardio-toxicity in rats. Rats were pre-treated orally with SA (50 mg/kg), RV (50 mg/kg) and combination of SA (25 mg/kg) and RV (25 mg/kg) along with positive control gallic acid (50 mg/kg) for 30 days. The effects of ISO on cardiac markers, lipid profile and lipid peroxidation marker, anti-oxidant enzymes and m-RNA expression of nuclear factor-kappa B (NF-kB) and tumor necrosis factor-α (TNF-α) were observed along with histopathological observations of simple and transmission electron microscopes (TEM). Serum creatine kinase-MB (CK-MB), lactate dehydrogenase (LDH) and alkaline phosphatase were significantly increased while cardiac tissue CK-MB, LDH, superoxide dismutase and catalase were significantly decreased in ISO administered rats, which also exhibited a significant increase in total cholesterol, triglycerides, low density lipoprotein cholesterol, very low density lipoprotein cholesterol and thiobarbutyric acid reactive substances and significant decrease in high density lipoprotein cholesterol in serum and heart. The m-RNA levels of inflammatory markers NF-kB and TNF-α were significantly increased in ISO treated rats. COMB Pre-treatment significantly reversed the ISO actions. Histopathological studies of simple and TEM were also co-related with the above biochemical parameters. Docking studies with NF-kB were also performed. Evidence has shown for the first time in this approach that COMB pre-treatment ameliorated ISO induced cardio-toxicity in rats and revealed cardio-protection.

## Introduction

A worldwide health problem, coronary artery disease (CAD), very often leads to myocardial infarction (MI). Among the patients of coronary heart disease, the principal reason for mortality worldwide is MI. As expected by World Health Organization, in 2020, most of the deaths will occur due to MI^1^. According to the Global Burden of Disease 2016 Study, cardiovascular diseases (CVD) alone accounted for 24% of total burden in men and 20% of total burden in women^2^. Insufficient blood supply to the myocytes leads to MI that brings about changes in electrical, mechanical, basic and biochemical properties of heart^3^.

Isoproterenol (ISO) is a synthetic β-adrenergic agonist used to induce acute MI^4^. The main mechanism of ISOinduced myocardial ischemia is free radicals production and reactive oxygen species (ROS), oxidative stress, lipid peroxidation (LPO) and overload of calcium (Ca^2+^) etc^5^.

Polyphenols are found in numerous plants and have anti-inflammatory and anti-cancer abilities, which can remove ROS that are formed by LPO^6^. Specifically, syringic acid (SA) was known to possess anti-diabetic^7^, hepato-protective^8^, anti-oxidant^9^, anti-endotoxic^10^, anti-steatotic, anti-inflammatory^11^, anti-hypertensive^8^, neuro-protective^12^ and anti-cancer activities^13^. SA has been reported for cardio-protection against ISOinduced cardio-toxicity by ameliorating pro-inflammatory cytokines in rats^14^.

Resveratrol (RV) is a characteristic phytochemical found in a wide variety of plant species including grapes, nuts and red wines^15^. Various exploratory investigations have proved that RV is involved in a few pathological pathways in various CVD, for example, MI^16^, myocarditis^17^, heart hypertrophy^18^.

Several conventional medicines have been investigated prior for the prevention and treatment of CAD, which are in the class of antagonist of Ca^2+^ and β-blockers. Plant originated drugs are more effective, safe and with no side effects. They play a crucial role in the prevention of MI, which can be done effectively with drugs originated from plants due to acting as potent anti-oxidants, which can reduce the generation of free radicals and thereby preserving the activities of anti-oxidant enzymes^19^.

Furthermore, the examinations utilizing in silico molecular docking tools give data linked to various phytochemicals with key proteins. This helps further to develop new therapeutic strategies to treat CVD. Hence, the present work was designed to investigate the interactions of SA, RV and combination of SA and RV (COMB) with key proteins with the help of in silico molecular docking studies.

Many side effects were associated with synthetic cardio-protective drugs. So there is a need to discover the drugs with no side effects. Mostly natural phytochemicals are beneficial in the prevention of diseases with no side effects. SA and RV are natural phytochemicals selected to test the protective effect on heart. Furthermore, combination therapy is often considered to be the best treatment for prevention of onset of several diseases. Here we performed the study with COMB for best effects along with individual phytochemicals (SA, RV) in ISO induced myocardial localized necrosis in rats.

## Materials and Methods

ISO, SA, RV, Gallic acid (GA) were purchased from Sigma Aldrich Company, USA. Total cholesterol (TC), high density lipoprotein cholesterol (HDL-c) kits were purchased from M/S Excel Diagnostics Pvt. Ltd., India except lactate dehydrogenase (LDH) kit was purchased from Proton Biological India Pvt. Ltd. Creatine kinase-MB (CK-MB) and triglyceride (TG) kits were purchased from Erba Company, India. Dimethyl sulfoxide (DMSO) was purchased from SDFCL, India while the primers of nuclear factor-kappa B (NF-kB) and tumor necrosis factor-α (TNF-α) were purchased from TaKaRa Corp, Japan. Patch dock server was used for docking studies while other chemicals, used were of analytical grade.

### Animals

Albino Wistar rats of male sex (100–120 g) were exposed to 7 days acclimatization to the conditions of animal house. Well ventilation, 25 °C temperature and 12/12 light and dark cycles were maintained carefully in animal house. Standard pellet diet and sufficient water were provided to rats. Sri Krishnadevaraya University, ananthapuramu, India obtained ethical clearance for conducting experiments on animals from Committee for the Purpose of Control and Supervision of Experiments on Animals (CPCSEA) (Regd. 1889/GO/Re/S/16/CPCSEA), and the work was approved by the Institutional Animal Ethics Committee (IAEC) with protocol Number Sri Krishnadevaraya university/Biochemistry/08/2017 (SKU/Biochem/08/2017), confirming that all experiments were performed in accordance with relevant guidelines and regulations.

### Experimental protocol

SA, RV doses were fixed by conducting preliminary dose dependent test. Two doses of SA and RV such as 25 and 50 mg/kg were screened in ISO administered rats. The dose 50 mg/kg of SA and RV were effective in reducing the elevated levels of serum cardiac markers. Hence the higher dose 50 mg/kg of SA and RV were selected in the current investigation.

GA was used as a positive control. The phenolic compound GA has been reported as cardio-protective against ISO-induced myocardial infarction in Wistar rats^20^. GA was also been reported as positive control in the earlier study^21^.

Total 42 rats were divided into 7 groups, each group with 6 rats.

Group I. Control rats

Group II. ISO rats (50 mg/kg bw)

Group III. SA treated rats (50 mg/kg bw)

Group IV. GA pre-treated rats (50 mg/kg bw) + ISO

Group V. SA pre-treated rats (50 mg/kg bw) + ISO

Group VI. RV pre-treated rats (50 mg/kg bw) + ISO

Group VII. COMB pre-treated rats (SA at 25 mg/kg bw + RV at 25 mg/kg bw) + ISO.

DMSO was used as solvent for SA and RV while water was used as solvent for GA. Group I rats were given DMSO via oral route for 30 days, while rats of group III, IV, V, VI and VII were subjected to oral pre-treatment with compounds respectively as showed in protocol through intragastric tube for 30 days. On 29^th^ and 30^th^ days of experiment period (with a gap of 24 hours), ISO was injected sub-cutaneously to rats of group II, IV, V, VI and VII.

### Sample collection

Sodium pentobarbital (35 mg/kg I.P.) was injected to all rats after 12 hours of second ISO administration and cervical dislocation method was used to sacrifice the rats. Collection of blood and hearts were done immediately. Serum was separated from the collected blood and hearts were washed with ice-cold physiological saline and homogenized then centrifuged to collect the supernatant for various biochemical investigations. Tissue and serum samples were refrigerated at −80 °C.

### Biochemical measurements

Measurement of cardiac marker enzymes such as CK-MB and LDH were done with Erba kit and Proton Biological India Pvt. Ltd respectively except alkaline phosphatase (ALP) was estimated by the method of Tietz NW^22^. Kits of M/S Excel Company were utilized for lipid profile estimation except ERBA kit was used for TG estimation. Calculation of very low density lipoprotein-cholesterol (VLDL-c) was done by the formula of VLDL-c = TG/5, while low density lipoprotein-cholesterol (LDL-c) = TC (HDL-c + VLDL-c) was used for calculating LDL-c. To estimate LPO marker, thiobarbutiric acid reactive substance (TBARS) level was taken by the method of Okhawa H^23^. The activities of catalase (CAT) and superoxide dismutase (SOD) were measured by the methods of Beers and Sizers^24^ and Marklund S and Marklund G^25^ respectively.

### Reverse transcriptase-polymerase chain reaction (RT-PCR)

Extraction of total RNA of heart tissues from all groups of rats was done with Trizol reagent (USA invitrogen). According to the (Promega corporation, USA) description of reverse transcription kit, synthesis of CDNA was carried out. TaKaRa corporation, japans’ multiplex polymerize reaction kit was utilized for the polymerization of c-DNA, with the following primers, β–actins (300 bp): Sense: 5′GCCCCTGAGGAGCACCCTGT3′; Antisense: 5′ACGCTCGGTCAGGATCTTCA3′, NFKB (500 bp): Sense: 5′ACGATCTGTTTCCCCTCATCT3′; Antisense: 5′TGGGTGCGTCTTAGTGGTATC3′, TNF-α (500 bp products): sense: 5′tcccaacaaggaggagaagt3′; antisense: 5′ccttgatgtctaagtacttg 3′. For annealing at 54 °C, cycling parameters were 30 s, PCR amplification for 30 cycles and the products (8 µl) were analyzed using 1.5% agarose gel by electrophoresis and finally Kodak (2.0) software was used for the determination of the product.

Quantification of RT-PCR was done with image J software (version 1.49) and expressed as percentage of control group.

### Histopathological examination

After scarification of all experimental rats, hearts were isolated. After isolation of hearts, hearts were immediately washed with ice-cold normal saline. Now all hearts were sectioned and fixed in 10% formalin and embedded in paraffin wax and sectioned at 5 µm thickness. Haematoxylin and Eosin (H&E) stain was utilized for staining the sections of heart tissues. Finally after staining of this heart tissue sections, these sections were carefully examined under the light microscope and photomicrographs were taken at 10x magnification.

Histopathological changes were categorized into 3 changes according to previous study^26^ such as (0) no changes; (+) less damage; (++) moderate damage and (+++) severe damage.

### Ultra structural studies of heart tissues by transmission electron microscopy (TEM)

0.1 M phosphate buffer (pH 7.2) was used for rinsing the tiny pieces of myocardial tissue. Around, 1-mm heart pieces were cut and promptly fixed into 2.5% ice cold glutaraldehyde in 0.1 M phosphate buffer (pH, 7.2) and kept at 4 °C for 12 h and post fixed with 1% buffered osmium tetroxide. TEM study was carried out by processing the heart tissues. 2% uranyl acetate and 0.2% lead acetate were used for staining the grids containing sections. Then, the heart segments were analyzed under a transmission electron microscope (4000x).

### Molecular docking studies

To investigate the molecular mechanism of RVl, SA and GA as anti-inflammatory agents in ISO induced MI rat models; we performed computational docking studies against NF-kB transcriptional protein. The docking was performed by using Patch Dock server and the output docking results were analysed by PyMOL visualization tool. The 3D structures of ligands (GA (CID370), SA (CID10742) and RV (CID445154), were retrieved from Pubchem database and their pdb files were generated by online smile translator server. The crystal structure of NF-kB (PDB ID: 3GUT) was retrieved from the Protein Data Bank (http://www.rcsb.org/). Prior to initiating the docking simulations, all non-protein molecules were removed, for any alternative atoms locations only the required location was retained and energy minimized before docking studies. All docking simulations in Patch Dock were performed by using default parameters. The docking results of ligands with NF-kB are represented in the form of docking scores and the best docked complex was screened by ranking of their docking scores.

### Statistical study

To perform the statistical study, results were subjected to Duncan’s Multiple Range (DMR) test by considering p value (p < 0.05). Data was expressed as means ± SD.

## Results

### Effect of COMB on cardiac marker enzymes

Table 1 shows the effect of COMB on cardiac marker enzymes such as CK-MB, LDH and ALP in heart tissue and serum. ISO injected rats showed a significant (p < 0.05) decrease in the cardiac tissue levels of CK-MB, LDH and ALP while a significant (p < 0.05) increase in serum CK-MB, LDH and ALP when compared to control rats. A significant (p < 0.05) increase and decrease in the levels of CK-MB, LDH and ALP in heart tissues and serum respectively were found in the rats, which were pre-treated with GA, SA, RV and COMB in comparison with ISO group. However COMB pre-treatment in ISO injected rats showed best amelioration of alterations in cardiac marker enzymes, when compared with SA and RV alone therapies. Rats, which were pre-treated with SA did not show significant (p < 0.05) difference with control rats.

**Table 1:** Effect of combination (COMB) of syringic acid (SA) and resveratrol (RV) on cardiac marker enzymes in serum and heart tissues of control and isoproterenol (ISO) treated rats.

parameters	CK-MB (IU/L)		LDH (U/L)		ALP (U/L)	
	Serum	Heart	Serum	Heart	Serum	Heart
**Groups**						
Control	311.3 ± 1.7^a^	81 ± 2.7^a^	708.6 ± 7.2^a^	385 ± 10.9^a^	148.6 ± 5.8^a^	169.63 ± 6.57^a^
ISO	607.0 ± 9.1^b^	49.6 ± 5.4^b^	1590.8 ± 6.5^b^	232.1 ± 12.2^b^	304.3 ± 10.4^b^	104 ± 5.22^b^
SA	310.6 ± 0.7^a^	81 ± 2.2^a^	706.8 ± 8^a^	385.5 ± 8.9^a^	145.8 ± 3.4^a^	171.2 ± 8.37^a^
GA + ISO	353.6 ± 5.7^c^	61.6 ± 1.5^c^	757 ± 17.6^c^	255.3 ± 2.6^c^	178.5 ± 10.6^c^	150 ± 2.88^c^
SA + ISO	351.1 ± 19.1^c^	65.6 ± 1.3^d^	731.3 ± 13.2^d^	270.1 ± 2.1^d^	158.6 ± 4.4^a^	156 ± 2.76^c^
RV + ISO	339 ± 22.8^c^	68.8 ± 1.3^d^	730.1 ± 15.4^d^	311.5 ± 10.6^e^	157.8 ± 27^a^	162.83 ± 3.43^a$^
COMB + ISO	330.1 ± 10.6^d*^	72.6 ± 1.3^e^	717.6 ± 15.5^d#^	367.8 ± 10.5 ^f^	156.8 ± 2.8^a^	164.16 ± 1.86^a^

Results are mean ± SD values with unusual superscript letters (a, b, c, d, e and f) significantly differ from one another (p < 0.05, DMRT). *Group is significantly not different with RV + ISO group, whereas ^#^group is significantly not different with control and SA groups. ^a,$^Group is significantly not different with SA + ISO group.

### Effect of COMB on lipid profile

Tables 2 and 3 demonstrate the impact of COMB on lipid profile in the serum and heart tissues. Significant (p < 0.05) elevated levels of lipid profile such as TC, TG, LDL-c, and VLDL-c while significant (p < 0.05) depressed levels of HDL-c were found in the ISO administered rats, when compared to control rats. Administration of GA, SA, RV and COMB for 30 days to rats, which were treated with ISO, exhibited significant (p < 0.05) depressed levels of TC, TG, LDL-c, VLDL-c and significant (p < 0.05) elevated levels of HDL-c, when compared to ISO alone injected rats. As compared with SA and RV alone pre-treatments, COMB interestingly decreased the cardiac and serum lipid levels in ISO administered rats. SA group displayed no significant (p < 0.05) change with control group.

**Table 2:** Effect of combination (COMB) of syringic acid (SA) and resveratrol (RV) on total cholesterol (TC), triglycerides (TG), high density lipoprotein-cholesterol (HDL-c), low density lipoprotein-cholesterol (LDL-c) and very low density lipoprotein-cholesterol (VLDL-c) in serum samples of control and isoproterenol (ISO) administered rats.

Parameters	Control	ISO	SA	GA + ISO	SA + ISO	RV + ISO	COMB + ISO
TC (mg/dl)	85.6 ± 1.3^a^	132 ± 1.8^b^	84.3 ± 1.3^a^	112.3 ± 5.6^c^	107 ± 3.9^d^	101.5 ± 1.7^e^	93 ± 3 ^f^
TG (mg/dl)	80.1 ± 3.7^a^	140.1 ± 1.2^b^	79 ± 1.2^a^	113.5 ± 12^c^	107 ± 3.9^c^	105.8 ± 3^d*^	101.5 ± 1.7^d#^
HDL-c (mg/dl)	29.1 ± 1^a^	22.8 ± 1.3^b^	29.6 ± 1.3^a^	25.56 ± 2.6^c^	25.8 ± 1^c^	27.1 ± 1.2^c^	28.5 ± 0.5^a^
LDL-c (mg/dl)	40.4 ± 2^a^	81.1 ± 2.2^b^	40.4 ± 1.2^a^	64.1 ± 8.5^c^	59.7 ± 3.6^c^	53.1 ± 3^d^	42.2 ± 2.5^a^
VLDL-c (mg/dl)	16 ± 0.7^a^	28 ± 0.2^b^	15.8 ± 0.2^a^	22.7 ± 2.4^c^	21.4 ± 0.7^c^	21.1 ± 0.60^d$^	20.3 ± 0.3^d@^

Results are mean ± SD values with unusual superscript letters (a, b, c, d, e and f) significantly differ from one another (p < 0.05, DMRT). ^*, $^ and ^#, @^ groups are not significantly different with SA + ISO group.

**Table 3:** Effect of combination (COMB) of syringic acid (SA) and resveratrol (RV) on total cholesterol (TC), triglycerides (TG), high density lipoprotein-cholesterol (HDL-c), low density lipoprotein-cholesterol (LDL-c) and very low density lipoprotein-cholesterol (VLDL-c) in heart samples of control and isoproterenol (ISO) administered rats.

Parameters	C	ISO	SA	GA + ISO	SA + ISO	RV + ISO	COMB + ISO
TC (mg/dl)	4.2 ± 0.17^a^	8.7 ± 0.04^b^	4.1 ± 0.15^a^	6.6 ± 0.15^c^	6.1 ± 0.06^d^	5.8 ± 0.03^e^	5.4 ± 0.1^e^
TG (mg/dl)	3.7 ± 0.02^a^	6.3 ± 0.13^b^	3.6 ± 0.07^a^	5.8 ± 0.03^c^	5.5 ± 0.04^d^	4.9 ± 0.09^e^	4.3 ± 0.04^f^
HDL-c (mg/dl)	2.32 ± 0.06^a^	1.16 ± 0.12^b^	2.36 ± 0.24^a^	1.33 ± 0.03^c^	1.50 ± 0.03^d^	1.80 ± 0.07^e^	2.26 ± 0.09^a^
LDL-c (mg/dl)	1.199 ± 0.14^a^	6.29 ± 0.14^b^	1.190 ± 0.23^a^	4.168 ± 0.19^c^	3.565 ± 0.06^d^	3.028 ± 0.10^e^	2.486 ± 0.13^f^
VLDL-c (mg/dl)	0.756 ± 0.00^a^	1.274 ± 0.02^b^	0.750 ± 0.00^a^	1.165 ± 0.00^c^	1.110 ± 0.01^d^	0.986 ± 0.018^e^	0.946 ± 0.00^f^

Results are mean ± SD values with unusual superscript letters (a, b, c, d, e and f) significantly differ from one another (p < 0.05, DMRT).

### Effect of COMB on LPO

**Figure 1:**
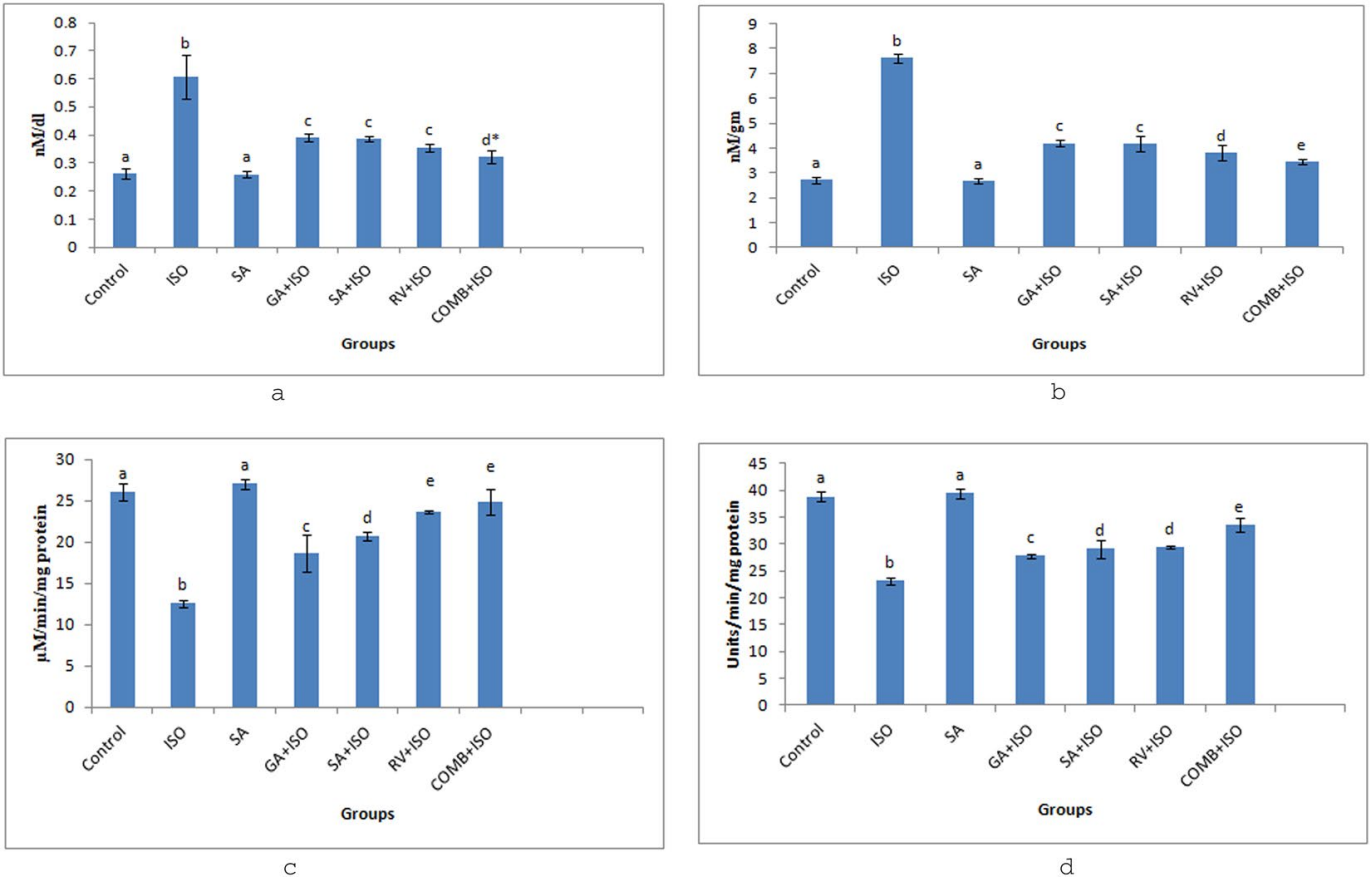
Effect of combination (COMB) of syringic acid (SA) and resveratrol (RV) on thiobarbutyric acid reactive substances (TBARS) in serum (**a**) along with heart tissues (**b**), catalase (CAT) in heart tissues (**c**) and superoxide dismutase (SOD) in heart tissues (**d**) of normal and isoproterenol (ISO) treated rats. Values are mean ± SD (n = 6 rats). Values that do not share a common superscript (**a**–**e**) differ significantly from each other (p < 0.05, Duncan’s multiple range test). *Group is significantly not different with RV + ISO group.

Figure 1a,b summarize the COMB effect on LPO in serum and heart respectively. Upon comparing with control rats, serum and heart tissues showed significant (p < 0.05) enriched TBARS levels in ISO group rats. However, after pre-treatment with GA, SA, RV and COMB the TBARS levels depleted significantly (p < 0.05) in ISO induced rats, when compared to ISO alone injected rats. Anyway COMB pre-treatment in ISO administered rats demonstrated best decrease in the LPO process, contrasting with SA and RV alone therapies. SA alone treatment significantly (p < 0.05) did not alter the TBARS levels, upon comparing with normal rats.

### Effect of COMB on anti-oxidant enzymes

Figure 1c,d show the effect of COMB on anti-oxidant enzymes such as CAT and SOD respectively in heart tissue samples. When compared with control group rats, ISO administered rats exhibited a drastic significant (p < 0.05) decrease in the levels of anti-oxidant enzymes such as SOD and CAT. Rats of COMB group showed significant (p < 0.05) increase in SOD and CAT levels after 30 days oral treatment with GA, SA, RV and COMB, when compared with ISO injected rats. COMB therapy in ISO operated rats shows better increase in these anti-oxidant enzymes, when compared to SA alone and RV alone pre-treatments. Significant difference was not observed in between control group and SA group rats.

### Effect COMB on m-RNA expression levels of NF-kB and TNF-α

**Figure 2:**
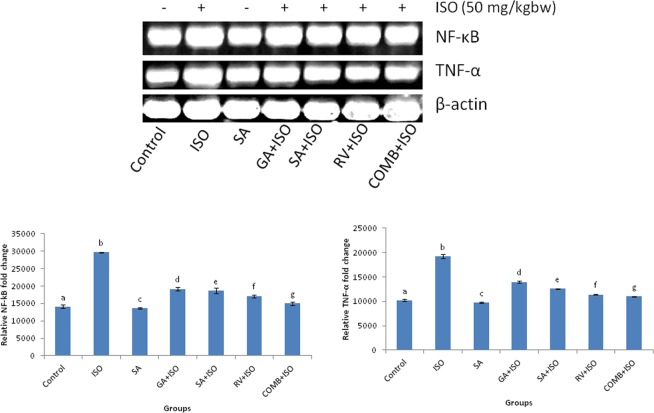
Effect of combination (COMB) of syringic acid (SA) and resveratrol (RV) on the expression levels of m-RNA of nuclear factor-kappa B (NF-kB) and tumor necrosis factor-α (TNF-α) of normal and isoproterenol (ISO) treated rats. Values are mean + SD (n = 6 rats). Values that do not share a common superscript (**a**–**g**) differ significantly from each other (p < 0.05, Duncan’s multiple range test).

Figure 2 represents the impact of COMB on NF-kB and TNF-α in heart tissues. Expression of m-RNA levels of NF-kB and TNF-α in heart tissues of ISO induced MI rats, were significantly (p < 0.05) augmented as compared with control group rats. Due to the oral treatment of GA, SA, RV and COMB to ISO administered rats for entire experimental period, significant (p < 0.05) decrease in the expression of m-RNA levels of NF-kB and TNF-α were found, upon comparing with ISO alone treated rats. Upon comparing SA and RV alone treatments, these m-RNA levels were significantly dropped with COMB therapy in ISO operated rats. SA alone therapy represented significant (p < 0.05) decrease in NF-kB and TNF-α expression, when compared to control rats.

### Effect of COMB on histopathological changes-simple microscope

**Figure 3:**
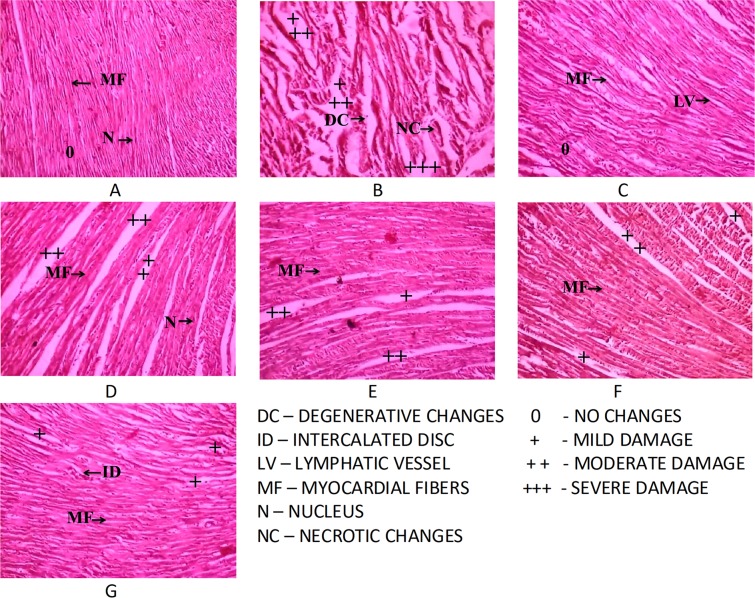
Photomicrographs of histopathological examination (H&E, 10x) of the heart tissues of normal, treated and isoproterenol (ISO) treated experimental rats. (**A**) Control group. (**B**) ISO group (50 mg/kg bw). (**C**) SA group (50 mg/kg bw). (**D**) GA + ISO group (50 mg/kg bw). (**E**) SA + ISO group (50 mg/kg bw). (**F**) RV + ISO group (50 mg/kg bw). (**G**) COMB + ISO group ((25 + 25) 50 mg/kg bw). (**A**) Photomicrograph of rat heart from control group showing normal architecture of heart with normal nucleus and usual myocardial fibers. (**B**) Photomicrograph of rat heart of isoproterenol-treated group showing focal myonecrosis and degenerative changes (arrows). (**C**) Photomicrograph of rat heart from syringic acid alone treated group showing normal architecture of heart with normal lymphatic vessels and myocardial fibers (arrows). (**D–F**) Photomicrographs of rat hearts of GA + ISO, SA + ISO and RV + ISO groups showing lesser degree of myonecrosis as evidenced by recovering myocardial fibres and lesser myocardial necrosis (arrows). (**G**) Photomicrograph of rat heart of COMB + ISO group showing almost recovered myocardial tissue as evidenced by almost usual intercalated discs and very lesser degree of myocardial necrosis and lesser number of damaged myocardial fibers than heart tissues of SA + ISO and RV + ISO groups.

Figure 3 indicates the effect of COMB on the extent of histopathological changes in myocardial tissues. Figure 3A illustrates the normal architecture of control rat heart in light micrograph. In ISO injected rats, severe alterations were found such as oedema, focal confluent necrosis of myofibres with infiltration of inflammatory cells, proliferation of fibroblasts & myophagocytosis along with RBC extravasations (Fig. 3B).

**Figure 4:**
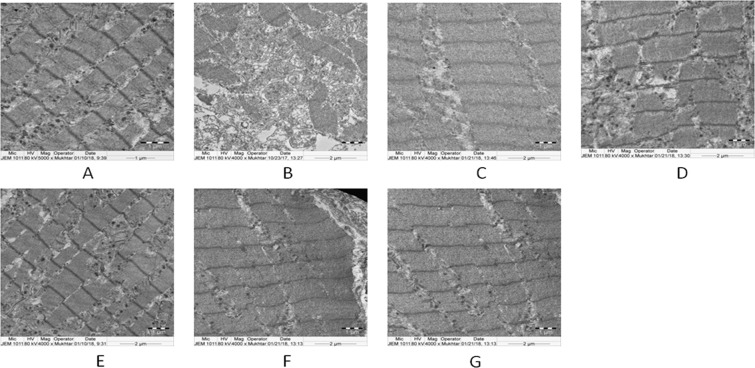
Depicts electron photomicrographs (TEM 4000x) of the heart tissues of normal, treated and isoproterenol (ISO) treated experimental rats. (**A**) Control group. (**B**) ISO group (50 mg/kg bw). (**C**) SA group (50 mg/kg bw). (**D**) GA + ISO group (50 mg/kg bw). (**E**) SA + ISO group (mg/kg bw). (**F**) RV + ISO group (50 mg/kg bw). (**G**) COMB + ISO group ((25 + 25) 50 mg/kg bw). Electron micrograph of heart of control rat showing regular arrangement of the myofibrils, mitochondria and regular Z lines. Electron micrograph of heart of ISO-induced rat showing disruption of myocardial fibers with swelling of heart mitochondria, irregular shape and size. Electron micrographs of a heart of GA + ISO, SA + ISO, RV + ISO and COMB + ISO treated rat showing nearly regular arrangement of the myofibrils, normal mitochondria and regular Z lines.

In GA + ISO (Fig. 3D), SA + ISO (Fig. 3E), RV + ISO (Fig. 3F) and COMB + ISO (Fig. 3G) groups, there was less oedema and myocardial necrosis with less inflammatory cells. However, COMB maximally recovered the damages caused to myocardial tissue by ISO administration, when compared to SA and RV groups individually. SA alone at the dose of 50 mg/kg bw administration did not lead to any histopathological changes in the myocardium (Fig. 3C). Damage scores to cardiac tissue of experimental rats are shown in Table 4.

**Table 4:** Effect of combination (COMB) of syringic acid (SA) and resveratrol (RV) on histopathological changes in heart tissue of control and isoproterenol (ISO) induced rats.

Histopathological changes	Control	ISO	SA	GA + I	SA + I	RV + I	COMB + I
Necrosis	0	+++	0	++	++	+	+
Inflammation	0	+++	0	++	++	+	+
Loss of transverse striations	0	+++	0	++	++	+	+
Myofibrillar degeneration	0	+++	0	++	++	+	+
Myophagocytosis	0	+++	0	++	++	+	+
Fibrosis	0	+++	0	++	++	+	+
Vacuolar degeneration	0	+++	0	++	+	+	+

No changes (0), mild damage (+), moderate damage (++) and severe damage (+++).

### Effect of COMB on structural alterations in heart tissues –TEM

Figure 4 exhibits the effect of COMB on ultra-structural alterations in heart tissues in normal and ISO group rats. TEM observation of ISO induced MI tissues showed irregular ‘z’- lines, fragmented myofibrils, mitochondrial swelling with alterations in shape and size, when compared with normal myocardial tissues. After treatment with GA, SA, RV and COMB, myocardial tissues revealed regular ‘z’- lines, nearly normal myofibrils and normal appearance of mitochondria. Above alterations were minimal in COMB group of rats than in individual pre-treatments in ISO administered rats. SA alone at the dose of 50 mg/kg bw administration did not lead to any histopathological changes in the myocardium.

### Docking studies of COMB with NF-kB

**Figure 5:**
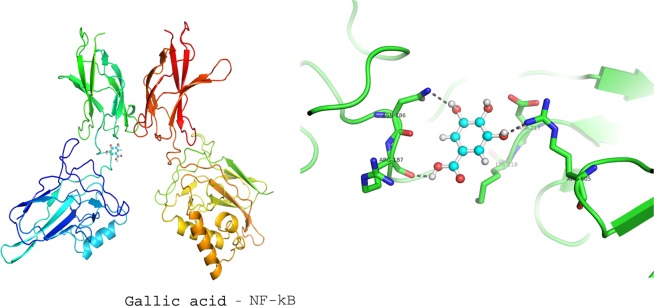
Displays the docking studies of gallic acid (GA) on nuclear factor-kappa B (NF-kB) in normal and isoproterenol (ISO) treated rats. Docking studies of NF-kB with GA shows 3 interactions with ARG 605, ARG 187 and ASN 186.

**Figure 6:**
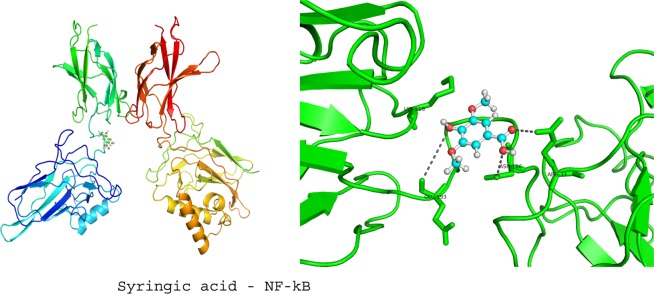
Displays the docking studies of syringic acid (SA) on nuclear factor-kappa B (NF-kB) in normal and isoproterenol (ISO) treated rats. Docking studies of NF-kB with SA shows 3 interactions with GLU 193, ASN 186 and ARG 33.

**Figure 7:**
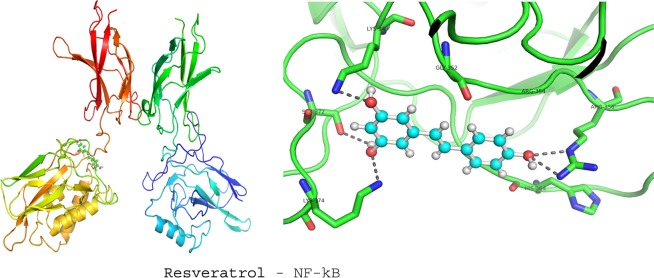
Displays the docking studies of resveratrol (RV) on nuclear factor-kappa B (NF-kB) in normal and isoproterenol (ISO) treated rats. Docking studies of NF-kB with RV shows 4 interactions with LYS 349, LYS 374, ARG 356 and SER 372.

**Figure 8:**
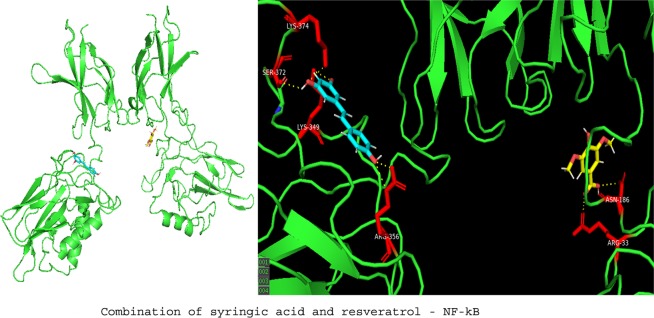
Displays the docking studies of combination (COMB) of syringic acid (SA) and resveratrol (RV) on nuclear factor-kappa B (NF-kB) in normal and isoproterenol (ISO) treated rats. Docking studies of NF-kB with COMB shows 6 interactions with ASN 186, ARG 33, LYS 349, LYS 374, ARG 356 and SER 372.

The docking score and binding interactions of ligands with NF-kB were shown in the Table 5. All ligands have shown hydrophobic, electrostatic and hydrogen bonding interactions (below 4 Å) with active site residues of NF-kB. Docking studies of GA, SA, RV and COMB were displayed in Figs. 5–8 respectively. In our docking study, we found that COMB obtained best docking score than individual docking studies with SA and RV alone. COMB interacted with ASN 186, ARG 33, LYS 349, LYS 374, ARG 356 and SER 372 residues whereas SA formed interactions with GLU 193, ASN 186 and ARG 33 and RV showed interactions with LYS 349, LYS 374, ARG 356 and SER 372 residues of NF-kB transcriptional protein.

**Table 5:** Docking studies of combination (COMB) of syringic acid (SA) and resveratrol (RV) on NF-kB receptor in heart tissues of normal and isoproterenol (ISO) induced rats.

S. No.	Receptor	Ligand	Interacted active site residues	scores	No of hydrogen bonds
1	NF-kB	Gallic acid	ARG 605, ARG 187 and ASN186	2348	3
2	NF-kB	Syringic acid	GLU 193, ASN 186 and ARG 33	2826	3
3	NF-kB	Resveratrol	LYS 349, LYS 374, ARG 356 and SER 372	3450	4
4	NF-kB	COMB(combination of syringic acid and resveratrol)	ASN 186, ARG 33, LYS 349, LYS 374, ARG 356 and SER 372	3786	6

## Discussion

There is an emergency to develop the safe and non-toxic cardio-protective agents, to eradicate the global burden of CVD especially MI. For this, the current investigation was designed to evaluate whether SA in combination with RV would have a synergistic cardio-protection against ISO-induced MI in rats.

Increase in the levels of CK-MB and other diagnostic marker enzymes in serum indicate the damage to cardiomyocytes, which is an indicative of MI. When ISO was injected, the levels of CK-MB, LDH and ALP increased in serum while decreased in the heart tissue. This might be expected that ISO induced changes in myocardial film integrity as well as permeability^27^, cardiac death and infarction^20^. These alterations in membrane may be due to free radicals attack and damage to myocardial membrane. Oral pre-treatment with GA, SA, RV and COMB to ISO administered rats, decreased the cardiac marker enzymes in serum and increased these diagnostic markers in heart tissues with maximum betterment in COMB + ISO group. It has been reported that oral pre-treatment of SA significantly protected against both ISO-induced cardio-toxicity^14^ and carbon tetra chloride-induced liver injury in rats^28^ by reducing the marker enzymes CK-MB, LDH, alanine transaminase and aspartate transaminase in serum due to the anti-oxidant activity of SA. Additionally, Feng *et al*.^29^ showed that RV ameliorated serum cardiac marker enzymes in ISOinduced MI rats by increasing the anti-oxidant enzyme SOD. Priscilla and Prince^20^ demonstrated that pre-treatment with GA in ISOinduced MI rats showed protective effect upon cardiac marker enzymes. The cardio-protection of COMB might be due to the conservation of the integrity and permeability of myocardial membrane by decreasing LPO and acting as anti-oxidant to scavenge free radicals or by increasing the activities of anti-oxidant enzymes in myocardial cells, thereby preventing free radicals attack on myocardial membrane.

ISO administration to rats is linked with increase in lipids, which finally leads to coronary heart disease^30^. TC, TG, LDL-c and VLDL-c were drastically elevated and HDL-c was demoted in rats, which were administered with ISO. These changes in lipid levels are because of improved lipid biosynthesis by elevated levels of cyclic adenosine monophosphate (cAMP)^31^. Lower levels of HDL-c and elevated levels of LDL-c also exhibit a positive correlation with MI. Jia Ren *et al*.^32^ and Wei-Jin Fang *et al*.^33^ showed that SA and RV treatment effectively decreased the lipid profile especially LDL-c and increased the HDL-C in diabetic and cardiomyopathy rats which is due to the anti-oxidant property of SA and RV. These facts are in line with our results, where Pre-treatment with GA, SA, RV and COMB demoted the levels of TC, TG, LDL-c and VLDL-c and elevated the levels of HDL-c. Surprisingly COMB decreased the lipid profile and increased HDL-c levels to near normal. This defensive impact of COMB is expected to the anti-hypercholesterolemic, anti-hyperlipidaemic and anti-oxidant impacts of COMB by decreasing the levels of cAMP, activating the lipoprotein lipase for the reduction of TC, TG levels, increasing the elimination of LDL and VLDL, increasing the synthesis of HDL-c and also decreasing the cholesterol synthesis by blocking the activity of 3-hydroxy-3-methyl-glutaryl-CoA reductase.

A mechanism of cellular damage which leads to the occurrence of MI is LPO. ISO administered rats showed augment in serum and heart tissue TBARS levels. ISO causes the production of highly reactive hydroxyl radical, which plays main role in the initiation of LPO^34^. Poly unsaturated fatty acids in the plasma membrane are the main targets for the attack of ROS, which form peroxy radicals, results in the occurrence of a cascade of reactions, elevating LPO^20^. Pre-treatment with GA, SA, RV and COMB decreased the LPO marker, TBARS in serum. COMB decreased the TBARS levels in rats with ISO treatment than SA and RV alone in ISO treated rats, which clearly reveal the anti-oxidant properties of COMB by decreasing the synthesis of ROS and increasing the anti-oxidant enzymes of myocardial cells. Our result is in line with previous report of Shahzad *et al*.^14^ who stated that SA treatment ameliorated ISO administered cardio-toxicity by the inhibition of LPO within the cardiac tissues of rats. On the other hand, Fang *et al*.^35^ showed that RV inhibited LPO in streptozotocin-induced diabetic cardiomyopathy in rats by improving mitochondrial function through PGC-1α deacetylation.

Regulation of oxidative stress was done by SOD and CAT, which are the first line of cellular defense system^33^. In ISO administered rats, decrease in the activities of anti-oxidant enzymes such as SOD and CAT were detected. Anti-oxidant enzymes are inactivated, when the superoxide and hydrogen peroxide levels are increased. This may be the major cause for decreased activities of these anti-oxidant enzymes^36^. Actually increase in superoxide and hydrogen peroxide occurs, when quinines are synthesized by ISO auto-oxidation and reaction of quinines with oxygen. This reveals that upset in oxidant-anti-oxidant balance by the formation of ROS in MI and will create the increased demand for anti-oxidant defense system. Pre-treatment with GA, SA, RV and COMB increased the activities of SOD and CAT in ISO treated groups. Anti-oxidant enzyme activities were significantly improved in rats of COMB + I group. This may be attributed to the role of COMB by protecting the anti-oxidant enzymes from the attack of free radicals. The presence of hydroxyl (OH) groups makes COMB as potent anti-oxidant. COMB pre-treatment may enhance the expression of these anti-oxidant enzymes, which will destroy the ROS, deacidifies quinonoids, detoxifies exogenous chemicals and increases synthesis of glutathione etc. Our results are in accordance with previous results of Shahzad *et al*.^14^ who stated that SA can scavenge ROS such superoxide and OH radicals by ameliorating the anti-oxidants such as CAT, SOD, reduced glutathioine, glutathione peroxidase and glutathione S-transferase. The results of Feng *et al*.^29^ showed that pre-treatment with RV significantly increased the activity of SOD in ISO injected rats. Moreover, Priscilla and Prince^20^ have demonstrated a cardio-protection of GA against ISO-induced MI, which was mediated through both enzymatic and non-enzymatic anti-oxidants.

Generation of inflammation is the critical factor in CVD. Attenuation of inflammation plays a significant role by anti-inflammatory agents. An increase in pro-inflammatory cytokines such as NF-kB and TNF-α with activation of oxidative pressure in myocardial damage leads to apoptosis and impairment of contractile capacity of the heart, sometimes even causes failure of heart^37^. The administration of ISO leads to elevated expression levels of NF-kB and TNF-α m-RNA. This might be due to the activation of NF-kB by ISO. Activation of NF-kB occurs via β-Adrenergic receptor dependent pathway by ISO^38^. In inflammation and apoptosis, NF-kB acts as a key regulator. In MI, phoshporylation of NF-kB induces pro-inflammatory cytokines such as TNF-α, IL-6, IL-1^39^. Pre-treatment with GA, SA, RV and COMB to ISO administered rats demoted the m-RNA levels of NF-kB and TNF-α. A significant decrease of these inflammatory m-RNA levels were found with COMB for entire experimental period in ISO administered rats. The main mechanism of cardio-protection is inhibition of inflammation by COMB which may be due to anti-inflammatory, anti-atherogenic and anti-oxidant activities of COMB. It has been reported that the anti-oxidant activity of SA exhibited cardio-protection in ISO administered rats^14^ and in streptozotocin-induced diabetic rats by regulating the inflammatory markers NF-kB and TNF-α^32^. RV attenuates inflammation in rat heart subjected to ischemia/reperfusion by regulating NF-kB and TNF-α^40^. Moreover, RV in combination with quercetin regulated the inflammatory marker TNF-α in acetaminophen-induced hepatocyte alterations by its anti-oxidant activity^41^.

Normal architecture was seen in the control rat’s hearts such as no inflammation, oedema. ISO administration leads to degenerative changes in hearts such as necrosis, interstitial oedema and infiltration of inflammatory cells. These pathological aberrations are due to insufficient supply of oxygen with paramount rise in the wall stress^42^, which illustrates that oxidative stress and inflammatory processes are involved in ISO-induced myocardial injury^43^. Necrotic changes occur by adrenochromes, which are formed through the auto-oxidation of ISO. During this process, free radicals are synthesized, which are detrimental to intra and extra cellular enzymes and proteins^44^. When pre-treated with GA, SA, RV and COMB reduced the degenerative changes in heart tissues, when compared to ISO alone administered rats. Necrosis, oedema and inflammation in heart tissues were significantly reduced with COMB pre-treatment in ISO challenged rats than individual therapies of SA and RV. Probably, this is related to necrotic preventive nature of COMB due to having the cyto-protective and anti-oxidant abilities of COMB.

Our TEM observations further supported the histopathological studies. The key features for heart diseases are loss of function of mitochondria and damage to mitochondria due to the generation of huge amount of free radicals by ISO administration. Changes in shape, size and swelling of mitochondria along with myofibril fragmentation were observed in heart tissues of ISO group. Reduced glutathione depletion leads to the accumulation of LPO products in mitochondria, which results in swelling of mitochondria. GA, SA, RV and COMB pre-treatment for 30 days to ISO induced rats showed ordered ‘Z’- lines, minimizations in mitochondrial swelling and regular arrangements of myofibrils. When compared with SA and RV alone, COMB intake leads to the minimization of alterations in shape, size and swelling of mitochondria and fragmentation of myofibrils in ISO injected rats. This may be due to scavenging of free radicals by showing anti-oxidant activity of COMB, thereby preventing from damage to heart tissue.

**Figure 9:**
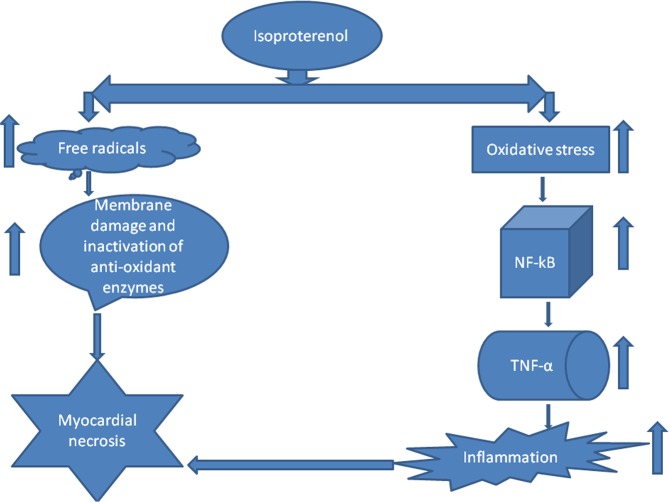
Shows the mechanism of induction of myocardial infarction (MI) by the administration of isoproterenol (ISO).

**Figure 10:**
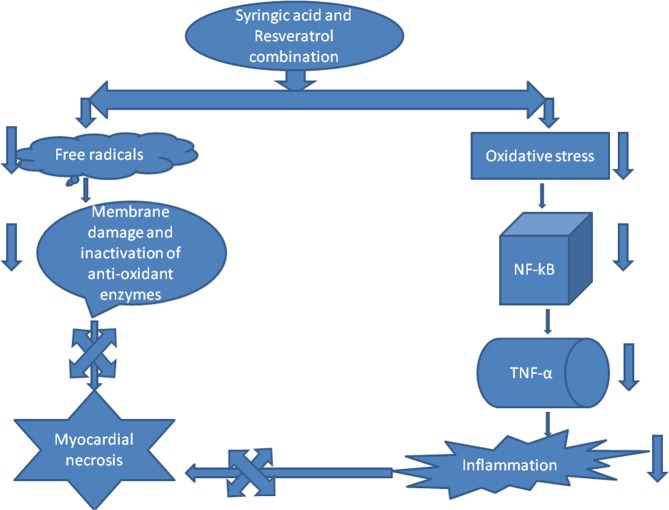
Exhibits the mechanism of cardio-protection by combination of syringic acid and resveratrol (COMB) against isoproterenol (ISO) induced myocardial infarction (MI).

In MI, inflammatory marker levels are increased. In our *in vivo* studies, we found that a marked decrease in the levels of NF-kB and TNF-α, after the pre-treatment with COMB. The results obtained in our *in vivo* studies are correlated with output of in silico studies. The test compounds (GA, SA, RV and COMB) have formed molecular complexes with the active site residues of NF-kB protein. The COMB has shown more docking score with NF-kB, when compared with individual docking studies. OH and carboxyl groups of ligands are sole players to make interactions with active site residues of NF-kB. Binding of ligands (GA, SA, RV and COMB) at NF-kB active site may interfere with the DNA interactions thus down-regulate/inhibit the NF-kB dependent gene regulation in inflammation. In our animal study, we found a drastic decrease in NF-kB and TNF-α levels in the heart tissues of rats, which were treated with COMB, when compared to SA and RV alone treated rats. We strongly suggest that our *in vivo* studies are greatly supported by the in silico studies and further we are planning to execute the detailed studies by applying molecular dynamics. Finally as depicted in Figs. 9 and 10 the cardio-protection was achieved effectively with the pre-treatment of COMB through the mechanism against ISO induced MI in albino wistar rats.

The current study for the first time demonstrates that SA and RV individually and COMB protected against ISO-induced MI in rats through NF-kB and TNF-α signalling pathways, which may provide new insights into the underlying mechanism of cardio-protection by COMB.

## Conclusion

Oral pre-treatment with COMB for a period of 30 days notably minimized alterations in cardiac marker enzymes, lipid profile, LPO, anti-oxidant enzymes, transcripts of inflammatory markers, histological alterations and finally docking studies with NF-kB than pre-treatments with SA and RV alone. Thus our examination reveals combination therapy is more effective than individual therapies, indicating the cardio-protective potency of COMB in ISO induced oxidative stress in rats.
